# MAD2B acts as a negative regulatory partner of TCF4 on proliferation in human dermal papilla cells

**DOI:** 10.1038/s41598-017-10350-w

**Published:** 2017-09-15

**Authors:** Nanlan Yu, Zhiqiang Song, Kezhou Zhang, Xichuan Yang

**Affiliations:** 1 Department of Dermatology, The First Affiliated Hospital of the Third Military Medical University, Chongqing, 400038 China; 2 Department of Dermatology, The 401st Hospital of Chinese People’s Liberation Army, 22 Minjiang Road, Qingdao, 266000 China

## Abstract

Dermal papilla cells (DPCs) are important components of hair follicles and play a critical role in hair follicle development. However, the mechanisms by which DPCs induce hair follicle development remain unclear. In the present study, we identified the mitotic arrest deficient protein MAD2B as a modifier of DPCs. Overexpression of MAD2B inhibited DPC aggregative growth and proliferation induced by the Wnt signaling activator T cell factor 4 (TCF4), and decreased TCF4-induced expression and the release of hair growth-related cytokines, including hepatocyte growth factor, insulin-like growth factor-1, and vascular endothelial growth factor in DPCs. In contrast, knockdown of MAD2B promoted TCF4-induced DPC proliferation, but did not affect the expression and secretion of cytokines by TCF4-induced DPCs. These results suggest a functional antagonism between MAD2B and TCF4 in DPC-induced hair follicle development. Mechanistically, MAD2B physically interacted with TCF4 to repress TCF4 transcriptional activity via β-catenin mediation, leading to reduced β-catenin/TCF4-dependent transactivation and Wnt signaling activity. These results demonstrate, for the first time, that MAD2B plays a negative role in TCF4-induced DPC growth and proliferation.

## Introduction

Human hair serves many functions, including thermal regulation, protection of the skin, sensory perception, as well as an aesthetic purpose^1^. Alopecia (hair loss) is a common chronic dermatological disorder that, although not life threatening or physically harmful, may result in psychological consequences such as anxiety and depression

^2^. Current medical treatments for hair loss include drug-induced hair growth promotion and hair transplantation, both of which require the formation of new hair follicles, from which hairs emerge^1^. Thus, a better understanding of the mechanisms underpinning the neogenesis of hair follicles is highly significant for the treatment of alopecia.

The hair follicle is structurally composed of epidermal and dermal portions^3^, and undergoes cyclical phases of growth (anagen), regression (catagen), and quiescence (telogen), throughout postnatal life^4,5^. Located at the base of the hair follicle, a population of mesenchymal cells, known as dermal papilla cells (DPCs), is characterized by aggregative behavior when cultured *in vitro* and the ability to induce new hair follicle formation during both embryonic and postnatal life^6–8^. DPCs promote hair follicle formation and development through the interplay of various signaling pathways such as Wnt, Bmp, Shh, and Notch^9,10^. Amongst these, Wnt/β-catenin signaling was the first identified and is considered the most important. Wnt/β-catenin signaling functions in hair follicle induction mainly through the binding of β-catenin to members of the lymphoid enhancer factor (LEF)/T cell factor (TCF) family of DNA binding proteins. This is triggered by Wnt-receptor interaction-induced β-catenin stabilization and translocation from the cytoplasm to the nucleus^11–14^.

In our previous study, TCF4, a member of the LEF/TCF family that is encoded by the Tcf7l2 gene, was found to be upregulated in DPCs in anagen hair follicles^15^. TCF4 and β-catenin form a complex in the nucleus, leading to transcriptional activation of downstream genes in the Wnt/β-catenin signaling pathway^16^. Furthermore, TCF4 is highly expressed in *in vitro* cultured low-passage (1‒3) DPCs, displaying an aggregative growth pattern that facilitates hair follicle induction. However, high-passage (>10) DPCs tend to lose their inductive ability during passaging^17–19^, suggesting that TCF4 is closely related to DPC proliferation and hair follicle neogenesis.

The mitotic arrest deficient protein MAD2B, a homolog of the cell cycle checkpoint protein MAD2^20,21^, has been identified as a TCF4-interacting partner. MAD2B blocks TCF4-mediated transactivation of the Wnt/β-catenin signaling cascade in the epithelial-mesenchymal transdifferentiation of colorectal cancer cells^22^. These findings prompted us to hypothesize that MAD2B/TCF4 interaction may suppress TCF4-induced DPC growth and proliferation by inactivating the Wnt/β-catenin signaling pathway. To test this hypothesis, we constructed adenoviral and plasmid vectors expressing MAD2B, TCF4, and short hairpin RNA against MAD2B. By transducing or transfecting these vectors, alone or in combination, into DPCs, we examined the role of MAD2B in TCF4-induced DPC growth and proliferation and explored the possible mechanisms involved.

## Results

### MAD2B inhibits the aggregative growth of DPCs

**Figure 1:**
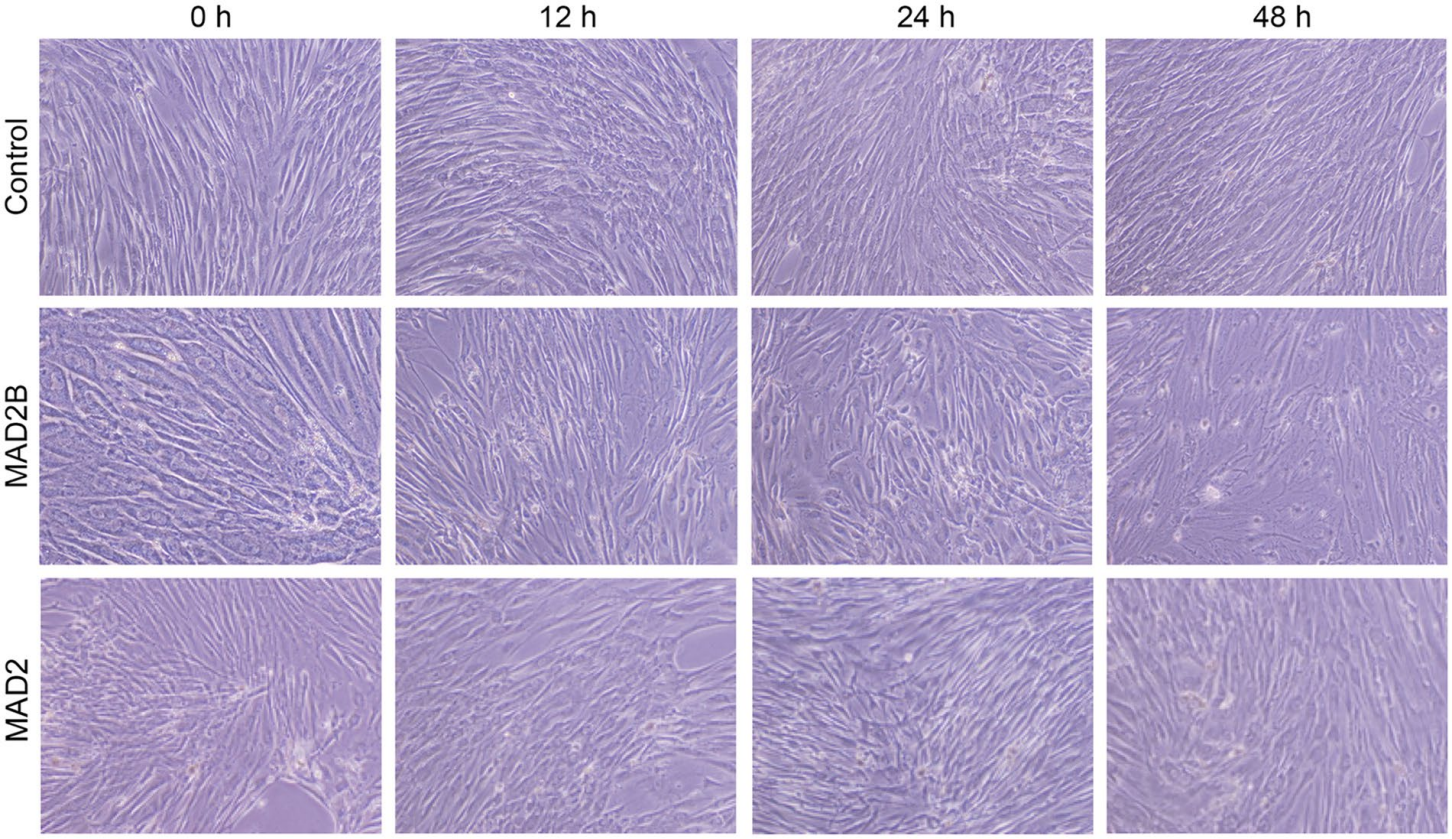
MAD2B overexpression suppresses the aggregative growth of DPCs. DPCs were transduced with a control vector (upper panels) or an adenoviral vector expressing MAD2B (middle panels) or MAD2 (lower panels) over time, as indicated. Micrographs were captured at 100 × magnification. DPCs, dermal papilla cells.

DPCs proliferate in a pronounced aggregative pattern *in vitro*, which has been shown to be important for their hair-inductive ability^18,23–26^. Furthermore, MAD2B plays a potential role in cell cycle arrest by inhibiting the anaphase-promoting complex (APC)^27^. To determine if MAD2B affects the aggregative growth of DPCs, an adenoviral vector expressing MAD2B was transfected into DPCs. As shown in Fig. 1, MAD2B-overexpressing DPCs exhibited a gradual loss of aggregative behavior in a time-dependent manner. After 48 h of transfection, the DPCs were loosely arranged and the aggregative behavior was clearly diminished (Fig. 1, middle panel). In contrast, the empty vector-transfected DPCs maintained the aggregative behavior and grew in clusters (Fig. 1, upper panel). Interestingly, MAD2 appeared to have no inhibitory effect on aggressive behavior of DPCs (Fig. 1, lower panel). These data suggest that MAD2B plays a specific role in inhibiting the aggregative growth of DPCs.

### MAD2B suppresses TCF4-induced DPC proliferation

**Figure 2:**
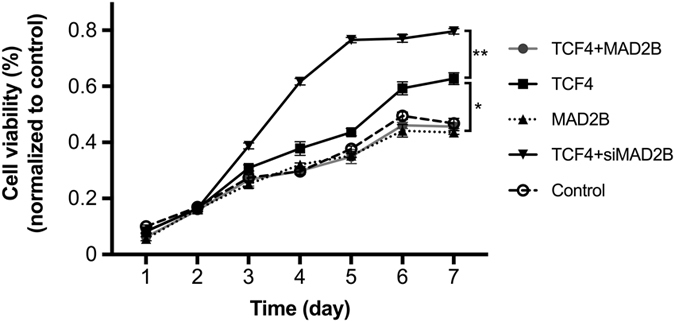
Effect of MAD2B on TCF4-induced DPC proliferation. DPCs were transfected with the vectors, as indicated, for 1‒7 d. The cell viability was determined through CCK-8 assays. The results shown are representative of three independent experiments. *P < 0.05, **P < 0.01 vs. control; *n* = 3. CCK-8, cell counting kit-8; TCF4, T cell factor 4; DPCs, dermal papilla cells.

Since our previous study indicated that TCF4 promoted DPC proliferation *in vitro*^19^, we next sought to examine whether MAD2B plays a role in TCF4-induced DPC proliferation. A CCK-8 assay was performed on DPCs transfected with TCF4- and MAD2B-expressing vectors and siMAD2B, individually or in combination. As shown in Fig. 2, the viability of DPCs transduced with TCF4 alone significantly increased in a time-dependent manner compared to the empty vector-transfected control group. However, the MAD2B-overexpressing DPCs proliferated at a comparable rate to control cells, suggesting that MAD2B has a minimal effect on DPC proliferation. Interestingly, DPCs co-transduced with TCF4- and MAD2B-expressing vectors exhibited a significantly lower proliferation rate than the DPCs transduced with TCF4 alone, suggesting that MAD2B has an inhibitory effect on TCF4-induced DPC proliferation. In contrast, DPCs co-transfected with TCF4-expressing vector and siMAD2B showed a dramatically higher proliferation rate than any other group, suggesting that TCF4-induced DPC proliferation can be further enhanced by knockdown of MAD2B. Taken together, these results indicate that MAD2B antagonizes TCF4 in DPC proliferation and point to the functional interaction between TCF4 and MAD2B in DPCs.

### MAD2B inhibits TCF4-induced expression and release of cytokines in DPCs

**Figure 3:**
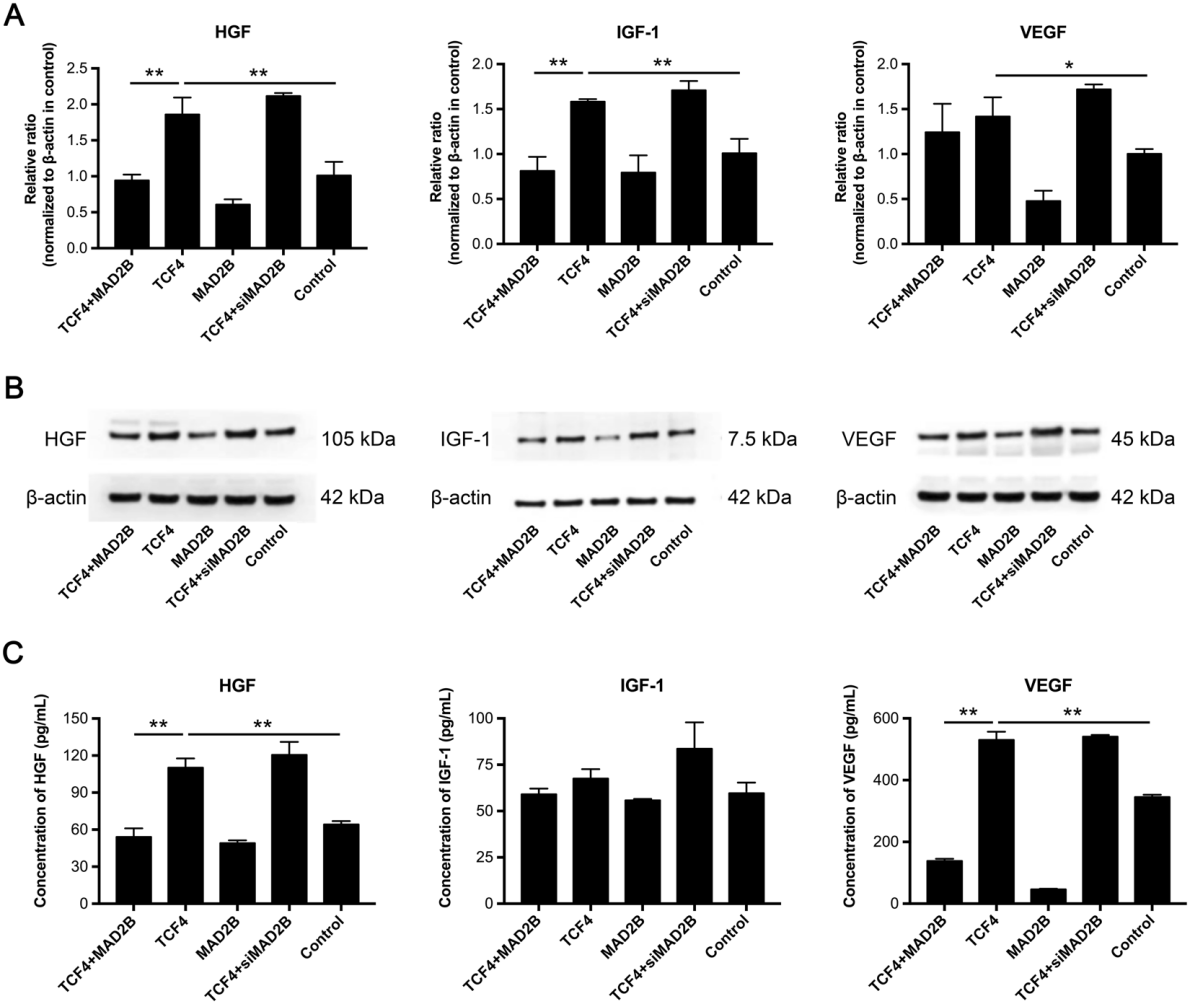
Effect of MAD2B on TCF4-mediated mRNA and protein expression and secretion of cytokines in DPCs. DPCs were transfected with the vectors, as indicated, for 24 h. Q-PCR, Western blot and ELISA were performed to examine the mRNA expression (**A**), protein expression (**B**), and secretion (**C**) of HGF, IGF-1, and VEGF, respectively. The results shown are representative of three independent experiments. *P < 0.05, **P < 0.01 vs. control; *n* = 3. TCF4, T cell factor 4; DPCs, dermal papilla cells.

To further confirm the functional interaction between MAD2B and TCF4, we examined the effects of MAD2B on the expression and release of certain hair growth-associated cytokines, including HGF, IGF-1, and VEGF, in TCF4-overexpressing DPCs. Consistent with the role of TCF4 in DPC proliferation, TCF4 overexpression promoted mRNA and protein expression of these cytokines in DPCs, which was effectively inhibited by overexpression of MAD2B (Fig. 3A and B). On the other hand, knockdown of MAD2B appeared not to markedly augment the cytokine-promoting effect of TCF4 (Fig. 3A and B), suggesting that MAD2B, while influential, is not essential to regulate TCF4 function. Furthermore, MAD2B suppressed the secretion of these cytokines by TCF4-overexpressing DPCs (Fig. 3C). Collectively, these results suggest that the function of TCF4 in cytokine production and secretion negatively correlates with MAD2B.

### MAD2B physically interacts with TCF4 in DPCs

**Figure 4:**
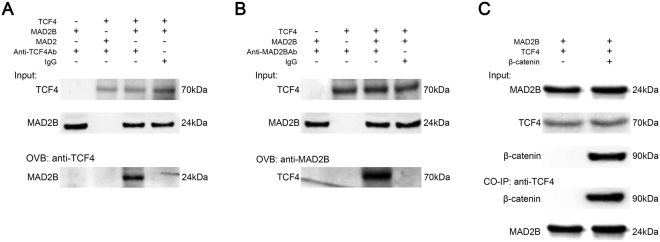
MAD2B physically interacts with TCF4. DPCs were transduced with the vectors, as indicated, for 24 h. Cell lysates were collected and immunoprecipitated with IgG (negative control), TCF4 antibody (**A** and **C**), or MAD2B antibody (**B**). The immunoprecipitates were blotted with MAD2B (**A**), TCF4 (**B**), and MAD2B/β-catenin (**C**). The results shown are representative of three independent experiments. IgG, Immunoglobulin G; TCF4, T cell factor 4; DPCs, dermal papilla cells.

Considering that the above findings indicate a functional interaction between MAD2B and TCF4, we next sought to examine whether MAD2B and TCF4 physically interact with each other. A Co-IP assay demonstrated that MAD2B, but not MAD2, was specifically bound to TCF4 in MAD2B/TCF4-co-overexpressing DPCs (Fig. 4A). A reverse Co-IP assay showed that TCF4 was specifically bound to MAD2B (Fig. 4B). These results suggest a physical interaction between MAD2B and TCF4, which is consistent with previous findings^22^. Furthermore, our results confirmed that overexpression of β-catenin, an essential component of the canonical Wnt signaling pathway, does not affect the interaction between MAD2B and TCF4. (Fig. 4C). Collectively, these data suggest that the ternary complex of MAD2B/TCF4/β-catenin may play an important role in DPC growth and hair follicle development.

### MAD2B is involved in the β-catenin/TCF4-mediated Wnt signaling pathway

**Figure 5:**
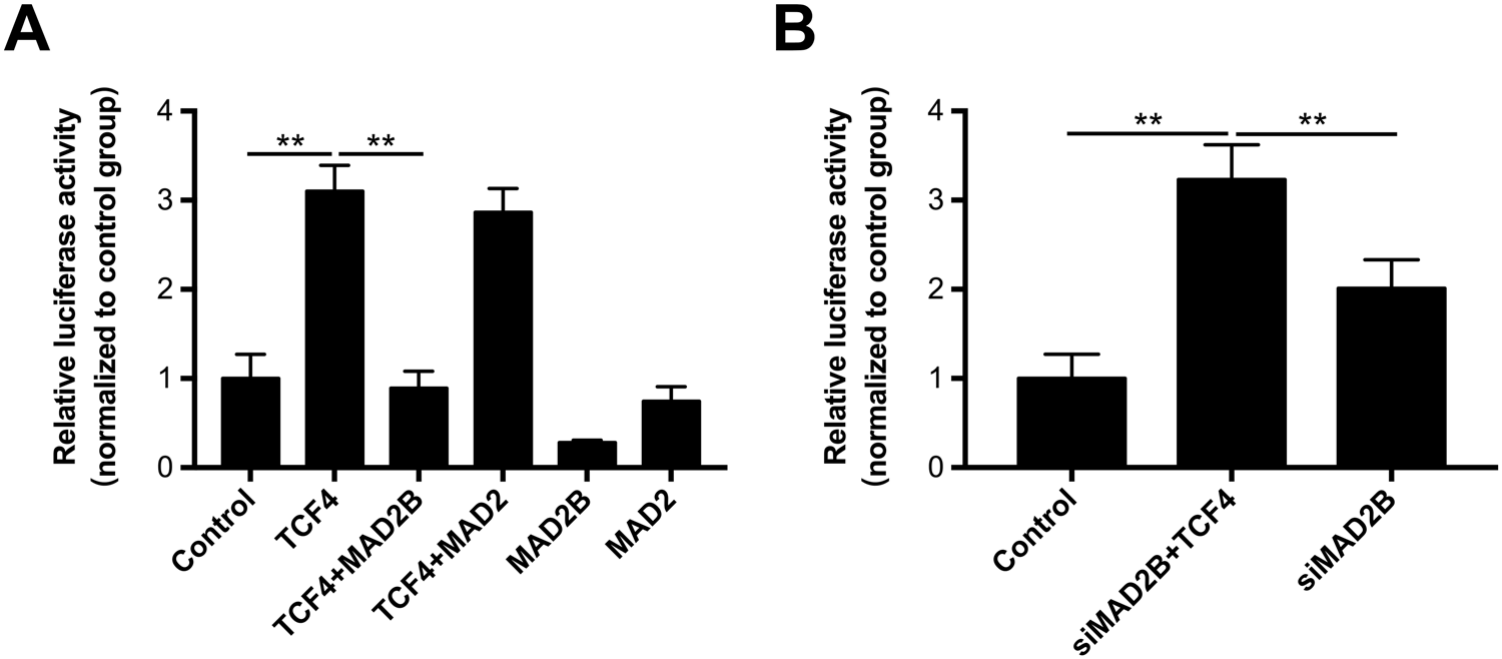
Effect of MAD2B on TCF4-mediated transactivation. (**A**) MAD2B suppresses TCF4-mediated transactivation through inhibiting TCF4 binding to DNA. DPCs were transfected with the vectors, as indicated. TOP-FLASH or FOP-FLASH was co-transfected into DPCs. Relative luciferase activity was determined 48 h after transfection and is shown as the TOP/FOP ratio. pRL-TK was also co-transfected as an internal control. (**B**) MAD2B knockdown increased TCF4-mediated transactivation. The results shown are representative of three independent experiments. **P < 0.01 vs. control; *n* = 3. DPCs, dermal papilla cells; TCF4, T cell factor 4.

To further investigate the molecular mechanisms underpinning DPC growth and proliferation, we performed a luciferase reporter gene assay. As shown in Fig. 5A, the luciferase activity was significantly higher in TCF4-overexpressing DPCs than in the empty vector-transfected control cells, suggestive of β-catenin/TCF4-enhanced transactivation or Wnt signaling activity. MAD2B, but not MAD2, inhibited the β-catenin/TCF4-induced upregulation of Wnt signaling activity. MAD2 appeared to have no effect on Wnt signaling activity regardless of the presence or absence of TCF4, suggesting that MAD2 is not involved in Wnt signaling. On the other hand, knockdown of MAD2B upregulated Wnt signaling activity, which was further promoted by TCF4 (Fig. 5B). These results suggest that MAD2B negatively regulates DPC proliferation and hair follicle development via the β-catenin/TCF4-mediated Wnt signaling pathway.

## Discussion

TCF4 is an important member of the LEF/TCF family. It has been reported that disruption of either TCF4 or β-catenin disrupts the Wnt signaling pathway, resulting in a complete loss of cell proliferation^28^. Our previous study has demonstrated that TCF4 is highly expressed in anagen hair follicles and cultured low-passage DPCs which are characterized by an aggregative growth pattern^15,19^, suggesting the important role of TCF4-β/catenin complex-mediated Wnt signaling in hair follicle morphogenesis. However, it remains largely unknown how TCF4/β-catenin transactivation is regulated during hair follicle induction and growth. In this study, MAD2B was found to suppress TCF4-mediated transcriptional activity in DPCs (Fig. 5). Overexpression of MAD2B inhibited the production and secretion of cytokines by DPCs (Fig. 3). Thus, MAD2B was identified as a negative regulatory partner of TCF4. However, knockdown of MAD2B did not affect the secretory functions of TCF4-induced DPCs (Fig. 3), but further promoted TGF4-induced DPC proliferation (Fig. 2). A possible explanation for this discrepancy is that the effect of MAD2B knockdown on cytokine production in DPCs may be counteracted by other factors.

MAD2B was initially identified as an inhibitor of the APC-cell division cycle 20 (CDC20) and APC-CDC20 homologue-1 (CDH1) complexation. Through direct interaction with CDC20 or CDH1, MAD2B can prevent the onset of anaphase^29^. CDC20 and CDH1 are essential regulators of mitotic cell division, leading to extensive inhibition of ubiquitination, necessary for the transition from metaphase to anaphase in the cell cycle. Intriguingly, it has been reported that CDC20 silencing can block Wnt/β-catenin signaling by preventing the negative regulator conductin from ubiquitination/degradation and subsequently increasing conductin/GSK3β-mediated phosphorylation/degradation of β-catenin^30^. Because MAD2B functions as a negative regulator of both TCF4 and CDC20/CDH1, it is possible that MAD2B plays a key role in the modulation of TCF4/β-catenin-mediated Wnt signaling in DPC growth and HF induction. This speculation is in agreement with the findings of the present study. DPCs are situated at the base of hair follicles, where they induce hair follicle neogenesis and control the cyclical growth of hair follicles^7,31^. These DPC properties have been exploited to develop therapeutic strategies for alopecia, including medical treatment and the surgical implantation of cultured DPCs^32,33^. It has been previously demonstrated that *in vitro* cultured DPCs exhibit aggregative behavior that is associated with their hair-inductive ability^7^. However, this growth pattern disappears gradually with increasing passage number. Due to the reduced mRNA and protein levels of TCF4 in high-passage DPCs^15,19^, a correlation between TCF4 expression and DPC aggregation behavior appears likely. In the present study, we first demonstrated that MAD2B can suppress the aggregative growth of DPCs *in vitro* (Fig. 1), which indicates that TCF4 and MAD2B have opposite effects on the aggregative behavior of DPCs, and gives us a clue that MAD2B may interact with TCF4 in DPCs to regulate DPC growth and hair follicle development. Many lines of evidence show that the aggregation growth of DPC is associated with versican, an extracellular matrix proteoglycan. Feng *et al*. reported that both mRNA and protein levels of versican decline along with a decrease in aggregative growth of high-passage DPCs, and RNA interference targeting versican suppressed DPC aggregation^34^. Furthermore, versican is found to be specifically expressed in DPCs during hair anagen, but downregulated during catagen and absent during telogen^35^. In addition, in vascular smooth muscle cells, TCF4-β/catenin complex can bind to the TCF binding sites in the promoter region of the versican gene, and versican transcription can thus be activated by the TCF4/β-catenin complex-mediated Wnt signaling pathway^36^. Further studies are required to determine whether MAD2B is involved in versican regulation.

It is well established that cytokines such as HGF, IGF-1, and VEGF promote hair growth^37–39^. HGF was first identified as a stimulator of epithelial cell dissociation and has been reported to activate hair follicle morphogenesis during the anagen phase^39,40^. IGF-1, a structural homolog of insulin, exerts insulin-like effects on glucose metabolism and plays a positive role in regulating hair follicle development^41^. The angiogenic growth factor VEGF promotes hair growth by inducing perifollicular angiogenesis during the anagen phase^42^. Consistent with these findings, the mRNA and protein levels and the secretion of these cytokines increase in response to TCF4 induction. These effects were diminished by overexpression of MAD2B (Fig. 3), suggesting a negative effect of MAD2B on the production and secretion of cytokines by DPCs.

In summary, the presented findings indicate that MAD2B suppresses the aggregation behavior of DPCs and inhibits TCF4-induced DPC proliferation and cytokine production by interacting with TCF4 and subsequently downregulating the TCF4-mediated Wnt/β-catenin signaling pathway. Thus, targeting MAD2B is a potential therapeutic strategy against alopecia.

## Materials and Methods

### Cell culture and transfection

This study was approved by The Ethics Committee of The First Affiliated Hospital of The Third Military Medical University, Chongqing, China. All experiments were performed in accordance with relevant guidelines and regulations. Full-thickness scalp skin samples were obtained from hospitalized male patients (aged 18‒35 years) undergoing plastic surgery. All patients provided written informed consent before sample collection. Primary culture of DPCs was performed as previously described^19^. DPCs were cultured in Dulbecco’s modified Eagle’s medium (DMEM) supplemented with 10% fetal bovine serum (FBS), penicillin (100 U/L), and streptomycin (100 U/L) at 37 °C in a humidified atmosphere of 5% CO_2_.

### Construction of plasmid and adenoviral vectors

Total RNA was extracted from human DPCs using Trizol reagent (Invitrogen, Carlsbad, CA, USA). The full length cDNA of MAD2B, MAD2, and TCF4 was synthesized with a reverse transcription kit (Toyobo, Osaka, Japan) using the following primers (Sangon, Shanghai, China): 5′-GGC CCA CTC CTC CCG GTA GC-3′ (forward) and 5′-GCG GGC GAT CCA CAC ACA GA-3′ (reverse) for MAD2B; 5′-CGC GTG CTT TTG TTT GTG TC-3′ (forward) and 5′-ATT TTC CTC ATG TCA TCC TCA GTC A-3′ (reverse) for MAD2; and 5′-TGG GGG TGA TTT TTT TTG GCT TT-3′ (forward) and 5′-GGG TTC ACG ACG CTA AAG CTA TTC T-3′ (reverse) for TCF4. Each cDNA was then cloned into a pIRES2 expression vector.

To generate an adenoviral vector expressing TCF4 (pAdTrack-CMV-TCF4), pIRES2-TCF4 was digested with *Hind III* and *Xba I* (Toyobo, Osaka, Japan), followed by ligation into pAdTrack-CMV (Addgene, Cambridge, MA, USA) using T4 DNA ligase (Toyobo). The resulting vector was transformed into DH10B competent cells (Takara, Dalian, Liaoning, China) and plasmid DNA was prepared using an E. Z. N. A. plasmid mini kit (Omega, Norcross, GA, USA), followed by recombination into the AdEasy adenoviral vector system (Addgene). The recombinants were transfected into 293 T cells using Lipofectamine 2000 (Invitrogen) for adenoviral packaging. The supernatant was collected for the preparation of adenoviral particles. pAdeno-MCMV-MAD2B expressing MAD2B was prepared by Genemine Biotechnology, Chongqing, China.

### Transduction and transfection

After DPCs were grown overnight to form a confluent monolayer, pAdTrack-CMV-TCF4 and pAdeno-MCMV-MAD2B were transduced into DPCs, individually or in combination, at a multiplicity of infection (MOI) of 50. plRES2-TCF4, plRES2-MAD2, and siMAD2B, a vector expressing short hairpin RNA against MAD2B that was constructed previously^22^, were transfected, individually or in combination, using Lipofectamine 2000 (Invitrogen), following the manufacturer’s instructions. The empty vector was used as a negative control.

### Evaluation of cell morphology and aggregation behavior

DPCs were seeded in a 6-well plate at a density of 3 × 10^5^ cells per well and incubated overnight. Cells were then transduced with pAdeno-MCMV-MAD2B or the empty vector and cell morphology was observed at 0, 12, 24, and 48 h under a light microscope (Olympus, Shinjuku, Tokyo, Japan) at 100 × magnification. The morphology and aggregation behavior of DPCs were imaged using an Olympus IX71 camera (Olympus).

### Cell proliferation assay

DPCs were plated into 24-well plates and grown overnight to form a confluent monolayer. Cells were then transduced with adenoviral vectors expressing TCF4 and MAD2B, individually or in combination, and siMAD2B was co-transfected with the TCF4-expressing vector. After 48 h of incubation, the transfected cells were harvested and plated in a 96-well plate at a density of 2 × 10^3^ cells per well. After cell attachment, 10 µL of Cell Counting Kit-8 (CCK-8) solution (Dojindo Molecular Technologies, Kumamoto, Japan) was added into each well, followed by dark incubation for 1.5 h. The absorbance [optical density (OD)] was determined at a test wavelength of 450 nm against a reference wavelength of 620 nm using a Varioskan Flash microplate reader (Thermo Fisher Scientific, Waltham, MA, USA). The cell viability (%) was calculated as (1 − OD_treated_/OD_control_) × 100%.

### Western blot analysis

DPCs were plated in 25-cm^2^ dishes at a density of 1 × 10^6^ cells per dish. The following day, cells were transfected with TCF4- and MAD2B-expressing vectors and siMAD2B, individually or in combination. After 48 h of incubation, cells were washed twice with cold phosphate-buffered saline, followed by cell lysis using radioimmunoprecipitation assay (RIPA) buffer (Beyotime, Shanghai, China). Cell lysates were collected and the protein concentration was measured using bicinchoninic acid protein assay reagent (Thermo Fisher Scientific). Subsequently, proteins (20 μg) were resolved by sodium dodecyl sulfate-polyacrylamide gel electrophoresis and transferred to polyvinylidene fluoride membranes (Bio-Rad, Hercules, CA, USA). The membranes were subsequently blocked with 5% nonfat milk in Tris-buffered saline containing 0.1% Tween 20, and then incubated with a primary antibody against β-actin, hepatocyte growth factor (HGF), insulin-like growth factor-1 (IGF-1), or vascular endothelial growth factor (VEGF) (R&D Systems, Minneapolis, MN, USA) for 1–2 h at room temperature. This was followed by incubation with horseradish peroxidase–conjugated secondary antibody for 1 h. After that, signals were detected using enhanced chemiluminescent substrates (Thermo Fisher Scientific). Images were acquired and quantified using a Bio2 Rad imaging system (Bio-Rad).

### Co-immunoprecipitation (Co-IP) assay

Cells were lysed with RIPA buffer (Beyotime) and the lysates were incubated with immunoglobulin G (IgG; negative control) or a monoclonal antibody against MAD2B or TCF4 (R&D Systems) using a Co-IP kit (Thermo Fisher Scientific). The immunoprecipitates were eluted and subjected to Western blot analysis, according to the manufacturer’s instructions.

### Luciferase reporter assay

DPCs were seeded in a 24-well plate at a density of 0.5 × 10^5^ cells per well and grown overnight. Cells were co-transfected with plRES2-TCF4, plRES2-MAD2B, and plRES2-MAD2, individually or in combination, and TOP-FLASH plasmid containing three TCF-binding sites or FOP-FLASH plasmid containing three mutated TCF-binding sites. Renilla luciferase plasmid (pRL-TK) was used as an internal control for normalization. After 48 h of incubation, cell lysates were prepared using luciferase lysis buffer (Promega, Madison, WI, USA). The luciferase activity was measured using a Varioskan Flash microplate reader (Thermo Fisher Scientific). Reporter activity is presented as the TOP-FLASH/FOP-FLASH ratio.

### Detection of intracellular and extracellular growth factors

DPCs were transfected with TCF4- and MAD2B-expressing adenoviral vectors and siMAD2B, individually or in combination. After 24 h of transfection, the culture medium was collected and total RNA was extracted from the cells using Trizol, followed by reverse transcription for cDNA synthesis. Quantitative polymerase chain reaction (q-PCR) was performed with the following primers: 5′-CAG CAG TCT TCC AAC CCA AT-3′ (forward) and 5′-CAC GAA CTG AAG AGC ATC CA-3′ (reverse) for IGF-1; 5′-CAG AGG GAC AAA GGA AAA GAA G-3′ (forward) and 5′-ATG CTA TTG AAG GGG AAC CAG-3′ (reverse) for HGF; and 5′-GTC CAA CTT CTG GGC TGT CT-3′ (forward) and 5′-CCC TCT CCT CTT CCT TCT CTT C-3′ (reverse) for VEGF. The concentrations of IGF-I, HGF, and VEGF in the culture medium were determined with commercial enzyme-linked immunosorbent assay (ELISA) kits (R&D Systems), following the manufacturer’s instructions. The absorbance (OD) was measured at 450 nm using a Varioskan Flash microplate reader (Thermo Fisher Scientific).

### Statistical analysis

All experiments were repeated at least three times. Data are expressed as the mean ± standard deviation (SD). Statistical analyses were performed using SPSS software, version 10.0 (SPSS, Chicago, IL, USA). A *P-*value of less than 0.05 was considered statistically significant.
