# Author Correction: MAD2B acts as a negative regulatory partner of TCF4 on proliferation in human dermal papilla cells

**DOI:** 10.1038/s41598-018-24989-6

**Published:** 2018-05-25

**Authors:** Nanlan Yu, Zhiqiang Song, Kezhou Zhang, Xichuan Yang

**Affiliations:** 1 Department of Dermatology, The First Affiliated Hospital of the Third Military Medical University, Chongqing, 400038 China; 2 Department of Dermatology, The 401st Hospital of Chinese People’s Liberation Army, 22 Minjiang Road, Qingdao, 266000 China

Correction to: *Scientific Reports* 10.1038/s41598-017-10350-w, published online 15 Spetember 2017

In the original version of this Article, Nanlan Yu and Zhiqiang Song were omitted as equally contributing authors.

In addition, there was an error in Figure 4, where the β-catenin results were listed as “−, −” instead of “−, +”. The correct Figure 4 now appears in the article.

This Article also contained errors in the Results section under subheading ‘MAD2B physically interacts with TCF4 in DPCs’.

“Furthermore, our results confirmed that TCF4 also physically interacted with β-catenin, an essential component of the canonical Wnt signaling pathway (Fig. 4C).”

now reads:

“Furthermore, our results confirmed that overexpression of β-catenin, an essential component of the canonical Wnt signaling pathway, does not affect the interaction between MAD2B and TCF4 (Fig. 4C).”

These errors have now been corrected in the PDF and HTML versions of the paper.

